# Supplementary material for: RG/RGG repeats in the C. elegans homologs of Nucleolin and GAR1 contribute to sub-nucleolar phase separation
Source: Nat Commun. 2022 Nov 3;13:6585. doi: 10.1038/s41467-022-34225-5 (PMC9633708; doi:10.1038/s41467-022-34225-5)

TITLE: RG/RGG repeats in the *C. elegans* homologs of Nucleolin and GAR1 contribute to sub-nucleolar phase separation

Supplementary Figure 1: **50 longest *C. elegans* RG/RGG repeats. a** Table of the 50 longest RG/RGG repeats in *C. elegans*. Proteins highlighted in blue are annotated to P-granules, yellow to the nucleolus, and green to stress granules. Proteins with an asterisk contain more than 1 of the longest RG/RGG repeats and appear in the table twice. **b** Locations of the 50 longest RG/RGG repeats within proteins.

Supplementary Figure 1: 50 longest *C. elegans* RG/RGG repeats

a

Protein Name	Length of RG/RGG domain	Location of RG/RGG within protein	Protein Name	Length of RG/RGG domain	Location of RG/RGG within protein
1.*T17H7.1	321	middle	26.M01G5.1	35	C terminus
2.K07H8.10	176	N terminus	27.Y94H6A.5	34	C terminus
3.LAF-1	135	N terminus	28.*RBM-12	34	middle
4.LPD-6	127	C terminus	29.*FIPR-21	33	middle
5.FIB-1	107	N terminus	30.F53C3.6	32	middle
6.*FUST-1	91	middle	31.SSB-1	30	middle
7.GRSP-4	79	middle	32.NLP-31	29	C terminus
8.*Y66H1A.4 (GARR-1)	70	C terminus	33.NLP-28	28	C terminus
9.PGL-3	66	C terminus	34.ZK792.5	28	middle
10.B0205.8	63	C terminus	35.GTBP-1	27	C terminus
11.F55A12.18	62	middle	36.NLP-30	26	C terminus
12.Y52B11A.11	58	middle	37.*T17H7.1	26	middle
13.*FUST-1	56	middle	38.*FIPR-21	26	C terminus
14.NLP-26	55	C terminus	39.LSM-4	25	C terminus
15.PGL-1	53	C terminus	40.SNR-3	25	C terminus
16.DRR-2	51	middle	41.F40F8.11	25	middle
17.*Y66H1A.4 (GARR-1)	48	N terminus	42.*HRPR-1	24	middle
18.RPS-2	48	N terminus	43.Y48C3A.14	24	C terminus
19.F13C5.2	45	middle	44.SFA-1	24	middle
20.MEK-5	44	C terminus	45.*CSR-1	24	middle
21.*HRPR-1	44	C terminus	46.*RBM-12	24	middle
22.NLP-32	42	C terminus	47.CEY-1	24	C terminus
23.RSP-6	38	middle	48.F53C3.3	22	middle
24.RHA-1	37	C terminus	49.C28C12.3	22	C terminus
25.NLP-29	36	C terminus	50.*CSR-1	21	N terminus

b

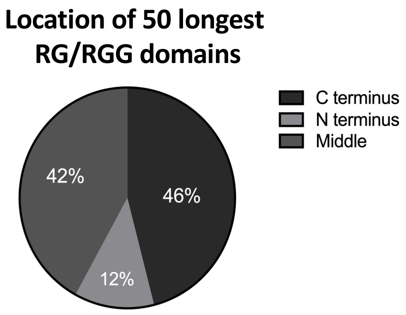

Supplementary Figure 2: **NCL homologs throughout evolution and deletion of NUCL-1. a** Phylogenetic tree of domain arrangement in NCL homologs. In most eukaryotes, NCL homologs contain an N terminal acidic region, middle RRMs, and a C terminal RG/RGG (black lines). Ecdysozoans rearrange these domains to an N terminal RG/RGG, a middle acidic region, and C terminal RRMs (blue lines).

Supplementary Figure 2: NCL homologs throughout evolution

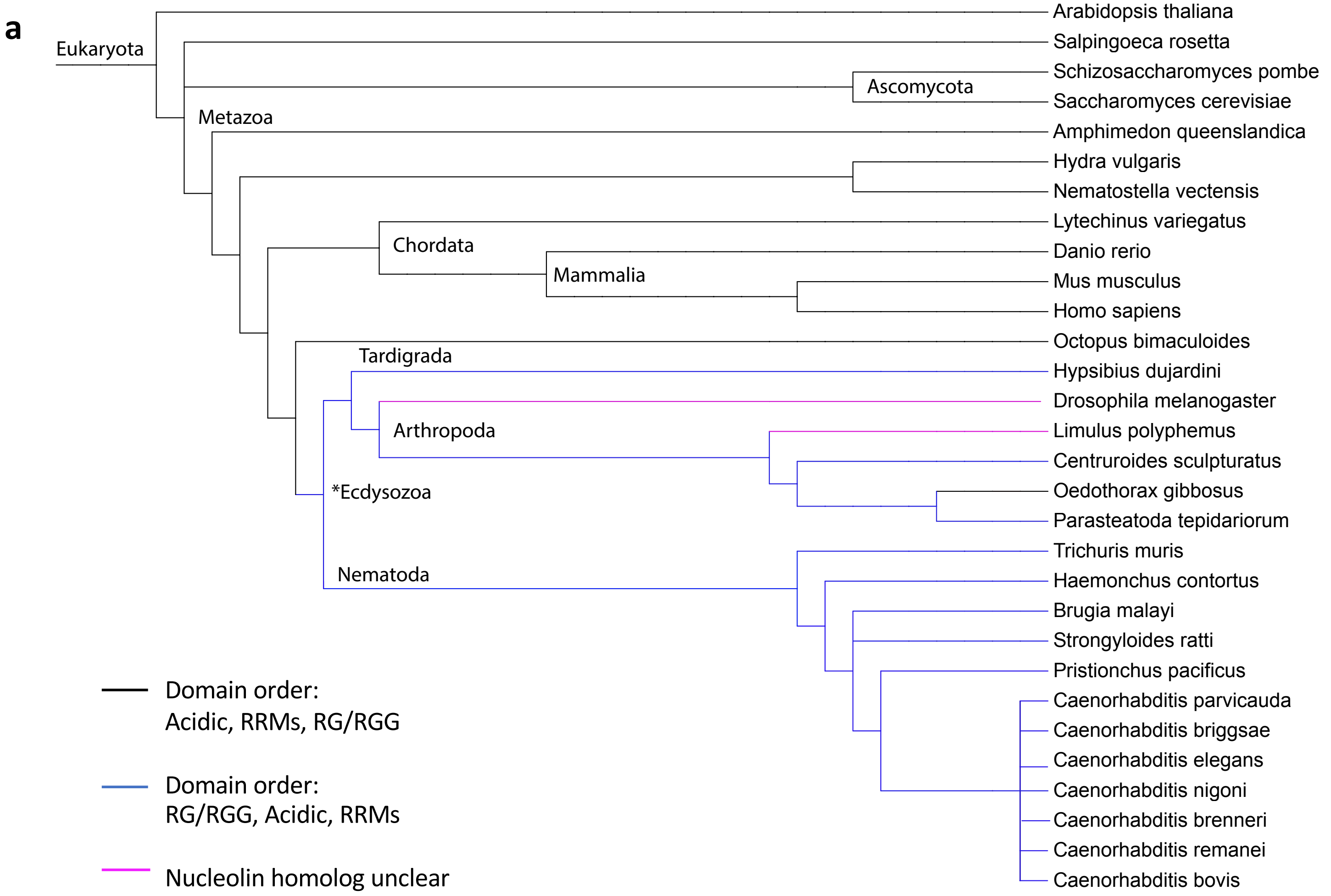

Supplementary Figure 3: **Nucleolar RG/RGG domain deletion and swapping. a** GLH-1::T2A::sGFP1-11 single oocyte. Upon T2A self-cleavage, sGFP1-11 is released from GLH-1 as a free molecule. The pink dotted circle represents the oocyte nucleus and the white dotted circle is the nucleolus. Free GFP does not organize into sub-compartments in nucleoli. **b** Western blot against the 16 amino acid sGFP11 peptide confirms the expected sizes of CRISPR/Cas9-edited NUCL-1 proteins. In ΔNUCL-1::sGFP11 worms the sGFP11 peptide is likely too small for detection (expected band size of linker + sGFP11 is ~3kDa). **c** Western blot against the 16 amino acid sGFP11 peptide confirms the expected sizes of CRISPR/Cas9-edited GARR-1 proteins. **d** Western blot against the 16 amino acid sGFP11 peptide confirms the expected sizes of CRISPR/Cas9-edited LPD-6 proteins. **e** GFP intensity of individual pachytene nucleoli in NUCL-1 WT vs NUCL-1 ΔRGG (n=94 WT and 88 ΔRGG nucleoli from 5 worms per genotype). **f** GFP intensity of individual nucleoli in GARR-1 strains (n=110 nucleoli from 6 WT worms, 67 nucleoli from 7 ΔNRGG worms, and 45 nucleoli from 5 ΔCRGG worms). Pachytene germ cell nucleoli from both GARR-1 RG/RGG deletion strains showed GFP intensity within WT range. GARR-1ΔNRGG showed a 16.5% decrease in the mean GFP intensity and GARR-1ΔCRGG showed a 12.1% decrease in the mean GFP intensity compared to WT. **g** GFP intensity of individual pachytene nucleoli in LPD-6 strains shows LPD-6ΔRGG::sGFP11 expression at or above WT levels (n=114 nucleoli from 7 WT worms and 108 nucleoli from 6 ΔRGG worms). **h** wrmScarlet (FIB-1) intensity of individual pachytene nucleoli in NUCL-1 WT and NUCL-1 ΔRGG worms (n=88 WT and 97 ΔRGG nucleoli from 5 worms per genotype). **i** GFP intensity of individual pachytene nucleoli in NUCL-1 WT, NUCL-1 ΔRGG, and NUCL-1(FIB-1RGG) (n=98 nucleoli from 5 WT worms, 90 nucleoli from 5 NUCL-1 ΔRGG worms, and 87 nucleoli from 6 NUCL-1(FIB-1RGG) worms). **e-i** Background-subtracted fluorescence intensity is shown. **e,g,h** Unpaired, two-tailed t-test with Welch's correction. **f,i** One-way ANOVA with Tukey's multiple comparisons test. **e-i** Data are shown as mean ± SD. *p<.05,****p<.0001.

Supplementary 3: Nucleolar RG/RGG domain deletion and swapping

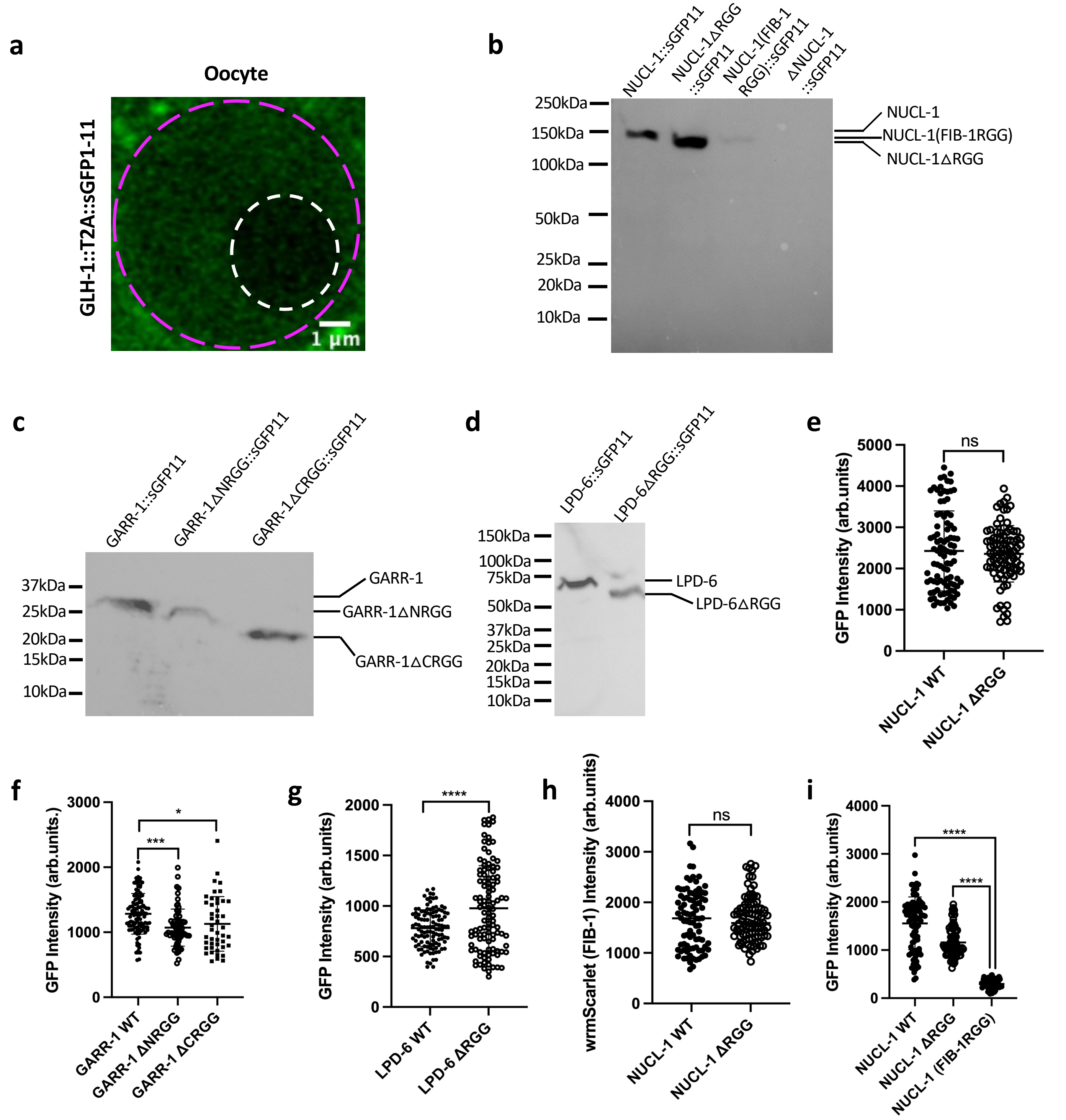

Supplementary Figure 4: **NUCL-1 and FIB-1 are still partially non-overlapping in NUCL-1ΔRGG worms. a** A straight line (white dashed line) was drawn across a single NUCL-1;FIB-1-labelled nucleolus and GFP and wrmScarlet fluorescence was measured. **b** A plot showing the opposite localization patterns of WT NUCL-1 and FIB-1. **c** A straight line (white dashed line) was drawn across a single NUCL-1ΔRGG;FIB-1-labelled nucleolus and GFP and wrmScarlet fluorescence was measured. **d** A plot showing that NUCL-1ΔRGG and FIB-1 still show areas of non-overlapping expression.

Supplementary Figure 4: NUCL-1 and FIB-1 are still partially non-overlapping in NUCL-1∆RGG worms

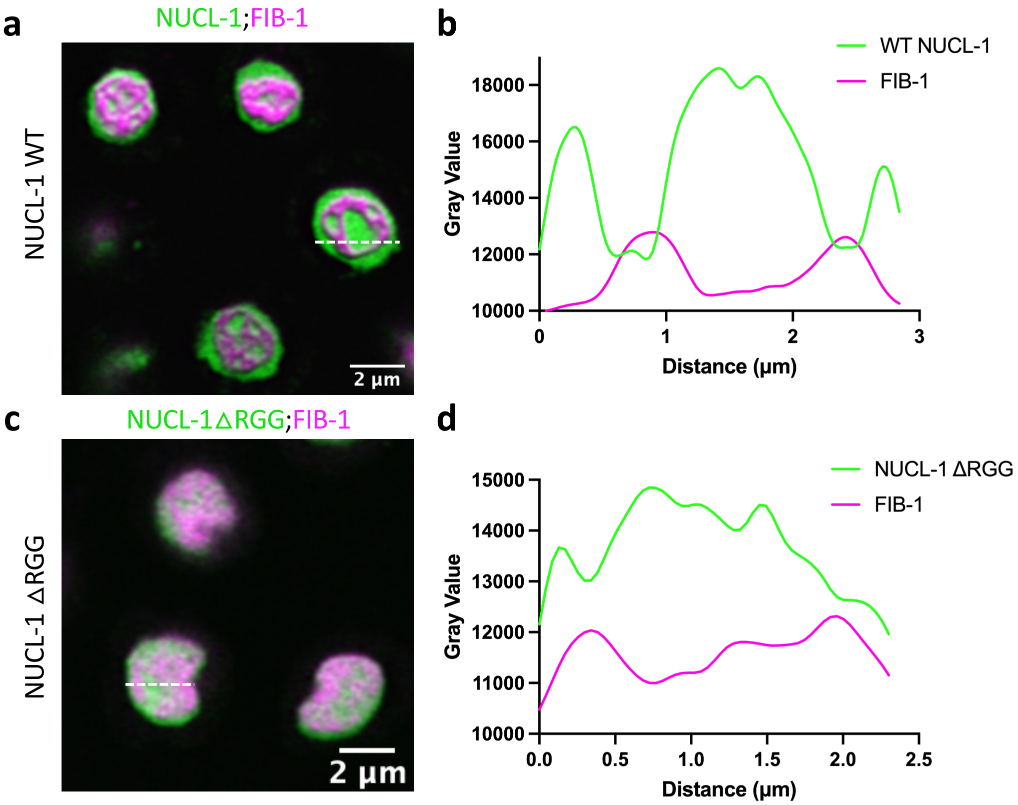

Supplementary Figure 5: **Full deletion of NUCL-1.** **a** Schematic of tagged WT NUCL-1 and deletion (ΔNUCL-1) and the genotyping primers used to confirm its deletion. **b** Gel image of PCR used to confirm ΔNUCL-1 using the primers shown in B. ΔNUCL-1 worms lack bands corresponding to the acidic and RRM domains. **c** Embryonic viability assay performed 5 generations post-NUCL-1 deletion shows no differences between NUCL-1 WT and ΔNUCL-1 in the percentage of hatched embryos 24 hours post-laying (n= 10 WT and 10 ΔNUCL-1 replicates). Unpaired, two-tailed t-test with Welch's correction. **d** Embryonic viability assay performed 40 generations post-NUCL-1 deletion still show no differences or delay in embryonic hatching. Both NUCL-1 WT and ΔNUCL-1embryos are 100% hatched 18 or 24 hours after laying (n= 6 WT and 6 ΔNUCL-1 replicates at each time point). **e** 40 generations after NUCL-1 deletion worms have subtle a decrease in brood size at 15°C (15°C: n=23 WT and 23 ΔNUCL-1 worms, 20°C n=22 WT and 22 ΔNUCL-1 worms, 25°C n=19 WT and 22 ΔNUCL-1 worms). 15°C p=.0116, 20°C p=.9985, 25°C p=.3652. **d,e** One-way ANOVA with Tukey's multiple comparisons test. **c,d** Points represent the percent fertility of 20 worms. **c-e** Data are shown as mean ± SD. *p<.05.

Supplementary Figure 5: Full deletion of NUCL-1

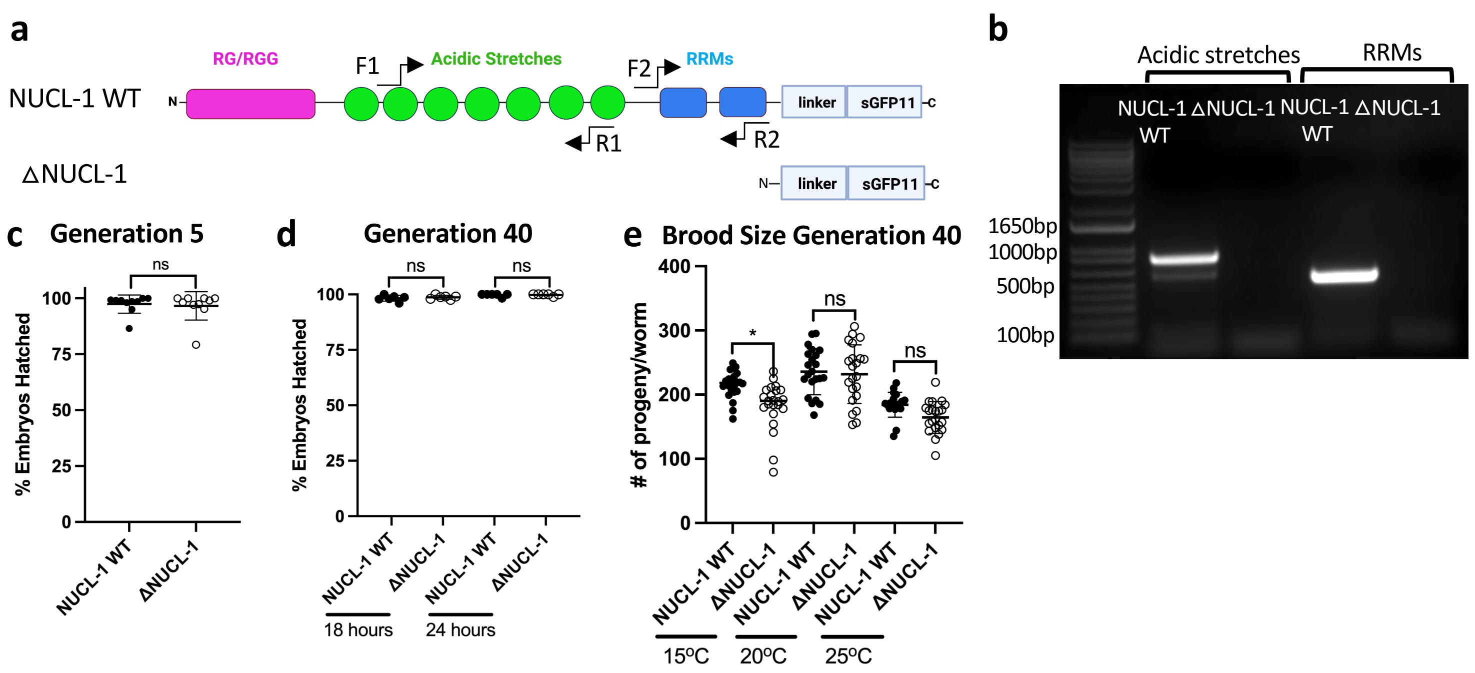

Supplement: Supplementary file 1 — Supplementary Information [file 41467_2022_34225_MOESM1_ESM.pdf]
