# Supplementary material for: RG/RGG repeats in the C. elegans homologs of Nucleolin and GAR1 contribute to sub-nucleolar phase separation
Source: Nat Commun. 2022 Nov 3;13:6585. doi: 10.1038/s41467-022-34225-5 (PMC9633708; doi:10.1038/s41467-022-34225-5)
Supplement: Supplementary file 3 — Description of Additional Supplementary files [file 41467_2022_34225_MOESM3_ESM.pdf]

Description of Additional Supplementary Files

File name: Supplementary Video 1
Description: 3- dimensional reconstruction of WT NUCL-1::sGFP;FIB-1wrmScarlet pachytene nucleolus. NUCL-1 (GC) surrounds and penetrates through FIB1 (FC).

File name: Supplementary Video 2
Description: Volume rendering of WT NUCL-1::sGFP;FIB1wrmScarlet pachytene nucleolus. NUCL-1 (GC) surrounds and penetrates through FIB-1 (FC).

File name: Supplementary Video 3
Description: Z stack of WT NUCL-1::sGFP;FIB-1wrmScarlet pachytene nucleolus. WT nucleoli show distinct compartmentalization of NUCL-1 in the GC and FIB-1 in the FC.

File name: Supplementary Video 4
Description: Z stack of NUCL-1DRGG::sGFP;FIB1wrmScarlet pachytene nucleolus. NUCL-1DRGG nucleoli show more homogeneous, but still partially nonoverlapping, NUCL-1 and FIB-1 distribution.
